# Zur Komorbidität von Posttraumatischer Belastungsstörung und Sucht in biopsychosozialer Perspektive

**DOI:** 10.1007/s40211-020-00384-4

**Published:** 2021-01-13

**Authors:** Hans-Peter Kapfhammer

**Affiliations:** Universitätsklinik für Psychiatrie und Psychotherapeutische Medizin, Medizinische Universität Graz, Auenbruggerplatz 31, 8036 Graz, Österreich

**Keywords:** Trauma, Posttraumatische Belastungsstörung, Substanzkonsumstörung, Komorbidität, Epidemiologie, Psychologie, Neurobiologie, Bindung, Trauma, Posttraumatic stress disorder, Substance use disorder, Comorbidity, Epidemiology, Psychology, Neurobiology, Attachment

## Abstract

Posttraumatische Belastungsstörung und Substanzkonsumstörungen treten im medizinischen Versorgungssystem häufig koexistent auf. Ihre Komorbidität geht mit schwerwiegenderen akuten klinischen Symptombildern, mit zahlreichen, oft notfallmäßigen Hospitalisierungen und geringeren Behandlungserfolgen einher. Ihre Komorbidität trägt zu dramatisch ungünstigeren Verläufen auf allen biopsychosozialen Ebenen bei. Das Thema Komorbidität von PTBS und Sucht wird auf mehreren Ebenen untersucht: in den Perspektiven von Epidemiologie, Substanzkonsumstörung als Risikofaktor für Trauma und PTBS, Trauma und PTBS als Risikofaktor für Substanzkonsumstörung, neurobiologischen Konsequenzen einer Substanzkonsumstörung für die Neurobiologie von PTBS, gemeinsam geteilten Faktoren der Genetik/Epigenetik, Persönlichkeitsdimensionen und aversiven/traumatogenen Einflüssen in der frühen Entwicklung. Der Hauptfokus der Analyse liegt auf den wechselseitig sich verstärkenden Mechanismen, die der Entwicklung und dem Verlauf beider Störungsbilder inhärent sind.

## Abstract (en)

Posttraumatic stress disorder and substance use disorder often co-occur within the health care system. Their comorbidity is associated with more serious acute clinical symptomatology, more frequent hospital admissions in state of emergency and significantly lower chances of improvement by psychological and pharmacological treatment. Their comorbidity contributes to dramatically unfavourable courses of illness as regards all biopsychosocial levels. The survey presented will discuss empirical findings from various perspectives: general epidemiology, substance use disorder as risk factor of trauma and PTSD, trauma and PTSD as risk factor of SUD, neurobiological effects of SUD converging towards neurobiology of PTSD, shared common factors of genetics/epigenetics, personality traits, and early developmental stress and trauma. The main focus of analysis will be put on processes that are intrinsically linked to the development and course of both disorders.

## Einleitung

Posttraumatische Belastungsstörung (PTBS) und Substanzkonsumstörungen treten im Versorgungssystem häufig koexistent auf und können hier große diagnostische und therapeutische Herausforderungen bereiten. PTBS wie Sucht stellen in einer biopsychosozialen Perspektive gleichermaßen komplexe psychische Störungen dar, die es zunächst je für sich kurz zu skizzieren gilt.

**Trauma** impliziert im psychologischen Verständnis die Konfrontation mit einer unausweichlichen Bedrohung der eigenen leiblichen Existenz. Traumatische Erlebnisse verweisen auf Extremsituationen des menschlichen Lebens. Sie unterbrechen den Lauf einer individuellen Biographie abrupt. Im Falle einer kollektiven Betroffenheit verändern sie gleichzeitig das Leben zahlreicher Mitglieder einer sozialen Gruppe signifikant. Konsequenzen nach Traumata sind sehr vielfältig. **Folgestörungen** nach einem Trauma sind aber keineswegs normativ. In einer klinischen Perspektive sind viele Faktoren wie prätraumatische Vorbelastungen und psychiatrische Vorerkrankungen, quantitative wie qualitative Dimensionen des äußeren aktuellen Traumas, Attribution subjektiver Bedeutungen und psychobiologisches Coping in der persönlichen Lebens- und Entwicklungssituation zu beachten. Aber auch Einflüsse der posttraumatischen Umwelt wie Verfügbarkeit und Güte der interpersonalen und sozialen Unterstützung sind zu berücksichtigen, um Ausmaß und Vielfalt der posttraumatischen Reaktionen, die Fähigkeit zur psychologischen und psychosomatischen Erholung wie auch das Risiko für eine chronische Beeinträchtigung besser verstehen zu können.

Die **Posttraumatische Belastungsstörung** (PTBS) stellt die prototypische, aber nicht die einzige Traumafolgestörung dar. Eine PTBS zeichnet sich bis zu DSM-IV-TR und im ICD-10 durch drei Symptomkomplexe aus, durch intrusiv auftretende Erinnerungen an das Trauma, durch eine systematische Vermeidung aller Trauma-bezogenen Aspekte sowie durch zahlreiche körperliche und kognitive Symptome einer autonom-nervösen Überaktivität aus. In DSM‑5 wird das klinische Bild einer PTBS breiter beschrieben. Es sind nunmehr auch negative Veränderungen in Kognitionen und Stimmungen in unmittelbarer Assoziation mit dem Trauma eigenständig abgebildet. Es werden hierüber dissoziative Erinnerungsstörungen, Depersonalisation/Derealisation und Gefühlsbetäubung, persistierende negative Überzeugungen oder Erwartungen an sich oder an die Umwelt, persistierende negative emotionale Zustände, Interessensverlust und Anhedonie erfasst. Eine der grundlegenden Erkenntnisse aus empirischen Studien ist, dass Traumata nicht nur dramatische **psychologische Verarbeitungsprozesse** auslösen, sondern auch tief in **biologische Regulationssysteme **eingreifen und funktionelle wie auch strukturelle Störungen im zentralen und autonomen Nervensystem nach sich ziehen können. Genetische Ausstattung, epigenetische Mechanismen und dispositionelle biologische Stresssysteme nehmen wesentlichen Einfluss darauf, ob eine spezielle traumatische Erfahrung zu schwerwiegenden psychischen Störungen (z. B. PTBS) und zu ernsten körperlichen Erkrankungsrisiken führt, oder aber mit einer erstaunlichen Kraft und Resilienz trotz anfänglicher Erschütterung gemeistert wird [92, 114].

**Substanzkonsumstörungen** werden heute ebenfalls als multifaktoriell bedingte, häufig chronisch verlaufende Störungsbilder definiert. Ein anthropologischer Blick auf „Sucht“ als existentielles Thema zwischen den Polen Rausch und Ekstase einerseits und Desintegration, Vereinsamung, Verzweiflung, Angst und Scham andererseits vermittelt wichtige Einsichten in das Grunddilemma eines süchtigen Menschen. Für ein tieferes klinisches Verständnis von Substanz-bezogenen und nicht-stoffgebundenen „Süchten“ in ihrer Heterogenität und Komplexität reicht dieser Zugang aber allein nicht aus. Wesentliche Voraussetzung für eine integrative Perspektive ist, „Süchte“ als eigenständige Krankheiten zu begreifen. Je unterschiedliche biologische, psychologische und soziale Einflüsse begründen eine individuelle Vulnerabilität. Diese Vulnerabilität manifestiert sich in akuten Konsumepisoden unter speziellen Entwicklungs- und belastenden Lebensbedingungen. Wiederum andere Faktoren können einen chronischen Verlauf fördern. Grundlegend ist: Suchterkrankungen führen häufig zu dramatischen nachteiligen Konsequenzen auf allen Ebenen des persönlichen, familiären und sozialen Lebens. Folgen aus Suchterkrankungen schließen zahlreiche psychische und somatische Komorbiditäten, den Verlust tragender psychosozialer Rollen, häufig einen sozialen Abstieg und vorzeitigen Tod mit ein [180].

Nachfolgend soll das Thema **Koexistenz/Komorbidität** von Trauma-bezogenen Störungen und Störungen durch Substanzkonsum aufgenommen werden. Ein spezieller Fokus soll hierbei auf mögliche inhärente Zusammenhänge von PTBS und Sucht gerichtet sein. Grundsätzlich sind hierbei mehrere Bedingungskonstellationen ins Auge zu fassen:

- Zufällige Koinzidenz von Substanzkonsumstörung und PTBS
- Substanzkonsumstörung versursacht direkt oder indirekt PTBS
- PTBS versursacht Substanzkonsumstörung
- Gemeinsame Ursachen für Substanzkonsumstörung und PTBS
- Substanzkonsumstörung und PTBS verursachen sich wechselseitig und interagieren nachteilig im weiteren Verlauf

Diese Zusammenhänge sollen nachfolgend aus mehreren Perspektiven anhand empirischer Befunde dargestellt und diskutiert werden.

## Epidemiologische Perspektive

Bereits erste epidemiologische Untersuchungen (Epidemiologic Catchment Area Studies) zeigten einen engen Zusammenhang von **PTBS** und **Substanzkonsumstörungen** in der Allgemeinbevölkerung, sowie besonders eindrucksvoll in störungsorientierten Behandlungskontexten. So bewegten sich die Prozentsätze von **koexistenter Suchtstörung** (Alkohol, Drogen) bei ehemaligen Angehörigen der US-Army mit der Diagnose einer PTBS in einem Bereich von 50–75 % [109]. Und umgekehrt wiesen Personen, die sich in Entwöhnungsprogrammen wegen Alkohol- oder Drogenproblemen befanden, in über 40 % auch eine **zusätzliche PTBS-Diagnose** auf [39]. Breit angelegte nachfolgende epidemiologische Surveys bestätigten diese signifikanten Assoziationen eindrucksvoll [151]. Sie spezifizierten auch nach einzelnen Substanzen.

In der für die US-amerikanische Bevölkerung repräsentativen Studie NESARC (National Epidemiological Survey of Alcohol and Related Conditions: *N* = 34.160) betrug die Lebenszeitprävalenz für **PTBS-allein, Alkoholabhängigkeit-allein** und **Koexistenz** von **PTBS und Alkoholabhängigkeit** je 4,83 %, 13,66 % und 1,59 %. Die **Komorbiditätsgruppe** zeichnete sich im Vergleich zu den beiden anderen Gruppen durch höhere Raten an frühkindlichen Traumatisierungen, durch häufigere Achse-I- und -II-Störungen, vermehrte Suizidversuche, einen früheren Beginn der PTBS, höhere PTBS-Symptomenanzahl, mehr Kriterien der Alkoholabhängigkeit, einen stärkeren zusätzlichen Drogenkonsum und gravierendere psychosoziale Behinderungsgrade aus [21]. In einer Detailstudie ebenfalls an diesem NESARC-Sample wurden Personen mit **Alkohol-Missbrauch/Abhängigkeit** sowie **mit** *versus ***ohne PTBS** hinsichtlich der prädiktiven Wertigkeit berichteter traumatischer Lebensereignisse (*n* = 27 distinkte Trauma-Typen) für eine inzidente Alkoholstörung untersucht. In einer multiplen Regressionsanalyse stellten sich für die Gruppe **mit koexistenter PTBS** statistisch signifikant erhöhte prädiktive Odds Ratios für die Traumatypen „*frühkindliches Trauma*“ (OR = 1,40; 1,08–1,83; *p* < 0,01) und „*gewaltsamer Übergriff*“ (OR = 1,41; 1,13–1,77; *p* < 0,01), aber auch für die Patientengruppe ohne PTBS signifikant erhöhte ORs für „*frühkindliches Trauma*“ (OR = 1,32; 1,23–1,41; *p* < 0,001), „*gewaltsamer Übergriff*“ (OR = 1,42; 1,13–1,78; *p* < 0,001), „*unerwarteter Tod eines nahen Angehörigen*“ (OR = 1,19; 1,12–1,28; *p* < 0,001) sowie „*Mitteilung eines Traumas bei einem nahen Angehörigen*“ (OR = 1,22; 1,13–1,30, *p* < 0,001) dar [61]. Systematische Reviews bekräftigten die enge Assoziation von Trauma, PTBS und Alkoholkonsumstörung sowohl für verschiedene Gruppierungen aus der Allgemeinbevölkerung als auch für die speziellen Populationen von Soldaten im aktiven Militärdienst und von Veteranen [44, 50, 74].

Systematische Reviews und Metaanalysen unterstrichen auch eine ähnlich starke Assoziation zwischen Trauma und PTBS einerseits und koexistentem Tabakkonsum (Zigarettenrauchen) andererseits. So betrug die durchschnittliche Prävalenz eines **aktuellen Tabakkonsums** bei Personen mit **PTBS **24 %. Umgekehrt lag die **koexistente** Diagnose einer **PTBS **bei **Zigarettenrauchern** in 20,2 % vor. Personen mit PTBS zeichneten sich zudem durch eine hochgradige Nikotinabhängigkeit und einen massiven aktuellen Konsum von Tabakprodukten aus [146]. Verglichen mit nichtrauchenden Personen aus der Allgemeinbevölkerung zeigten rauchende Personen eine ca. doppelt so hohe Rate an koexistenter PTBS. An PTBS leidende Raucher waren im Vergleich zu Rauchern ohne PTBS durch eine höhere negative Affektivität, eine umfangreichere Trauma-Anamnese, eine stärker ausgeprägte Nikotinabhängigkeit, häufigere andere psychiatrische Komorbiditäten, eine höhere Anzahl frustraner Abstinenzversuche und vermehrte Rückfälle nach zuvor schon erreichter Abstinenz von Nikotin charakterisiert [95].

Auch für die **Opiatabhängigkeit** betonten epidemiologische Untersuchungen einen überzufällig engen Zusammenhang zu Trauma und PTBS. Während Surveys für die US-amerikanische Allgemeinbevölkerung in ca. 60 % der Personen mindestens ein schwerwiegendes Trauma und eine durchschnittliche PTBS-Häufigkeit von 7 % in der Lebenszeitspanne aufdeckten, lag die Prävalenz von bedeutsamen **Traumata **bei opiatabhängigen Personen über 90 %, die in ca. 40 % der Fälle mit einer **koexistenten PTBS** einhergingen [128]. In der repräsentativen NESARC-Studie betrugen diese Prävalenzraten von PTBS und Opiatkonsumstörung je für sich 4,7 % bzw. 4,3 %. Hinsichtlich einer Komorbidität waren drei Befunde herauszuheben: Die vielfach adjustierte Odds Ratio bei Personen **mit PTBS** im Vergleich zu Personen **ohne PTBS** bezüglich koexistenter Opiatkonsumstörung war mit adj. AOR = 1,8 statistisch signifikant erhöht. Unter den Personen mit Opiatkonsumstörung (4,3 %) wiesen nur 0,7 % keine koexistente PTBS auf. Der Einfluss von **chronischen Schmerzsyndromen** bei PTBS-Personen war als signifikante Mediatorvariable für ein koexistentes Opiatkonsumrisiko besonders hervorzuheben (Muskelschmerzen: 31 % – adj. OR = 4,2; *p* < 0,001; neuropathische Schmerzen: 25,2 % – adj. OR = 3,1; *p* < 0,001; abdominelle Schmerzen: 12,8 % – adj. OR = 1,3; n. s. [18]). Eine Koexistenz von PTBS und Opiatkonsumstörung prädizierte mehrheitlich einen höheren Schweregrad des **Krankheitsverlaufs** sowie bedeutsamere Einbußen in der Gesundheits-bezogenen **Lebensqualität **[58, 73].

In einem epidemiologischen Fokus auf **suchtspezifische Behandlungseinrichtungen** stellte sich für Patient*innen mit zusätzlicher Diagnose **PTBS/Depression** auch eine überproportional erhöhte Assoziation mit einem **Mehrfachsubstanzkonsum **dar [32, 113].

Epidemiologische Analysen der **zeitlichen Abfolge** der koexistenten Störungen hoben mehrheitlich eine vorausgehende PTBS und eine nachfolgende inzidente Substanzkonsumstörung hervor [30, 31, 46, 199]. Aber auch die umgekehrte zeitliche Sequenz von vorausgehender Substanzkonsumstörung und nachfolgender inzidenter PTBS wurde in einigen Studien angedeutet [44, 50, 87, 176]. Die Assoziation zwischen **Trauma** und erhöhtem Substanzkonsumrisiko schien vorrangig über den **diagnostischen Status einer PTBS** vermittelt zu werden, konnte aber nach bedeutsamen, vor allem kumulativen Traumata auch ohne inzidente PTBS auftreten ([9, 30, 47, 61, 114, 152, 199].

Auf den prägenden nachteiligen Einfluss von **Traumata in frühen Entwicklungsabschnitten** nicht nur für zahlreiche psychische, Verhaltens- und Substanzkonsumstörungen, sondern auch für eine Reihe von somatischen Erkrankungsrisiken im Erwachsenenalter ist gesondert hinzuweisen. Mehrere prospektive Longitudinaluntersuchungen haben diesen Befund nachhaltig bestätigt. Ein systematisches Review mit Metaanalyse fand für Personen mit ≥ 4 aversiven oder traumatischen Belastungen in der Kindheit (ACEs) im Vergleich zu Personen ohne ACEs durchgängig signifikant erhöhte Risiken für alle untersuchten psychischen Störungen und somatischen Krankheiten im Erwachsenenalter. Die einzelnen Odds-Ratios stellten sich differentiell dar [82]:

- OR: < 2: körperliche Inaktivität, Übergewicht, Adipositas, Diabetes mellitus
- OR: 2–3: Rauchen, bedeutsamer Alkoholkonsum, schlechter selbst-beurteilter Gesundheitszustand, Karzinome, Herzkrankheit, respiratorische Krankheit
- OR: 3–6: riskantes Sexualverhalten, psychische Morbiditäten (Depression, Angst, PTBS), problematischer Alkoholkonsum
- OR: > 7: problematischer Drogenkonsum, selbst-verletzendes Verhalten/Suizidalität, interpersonale Aggressivität/Gewalt

Mehrere Studien replizierten diese signifikante pathogenetische Rolle von **frühkindlichen Trauma-Expositionen **für die Vermittlung einer **Koexistenz/Komorbidität** von **PTBS** und **spezieller Substanzkonsumstörung im Erwachsenenalter** [61, 113, 115, 130, 156, 214]. Diese engen Assoziationen dürfen jedoch nicht in einem Modell linearer Kausalrelationen konzeptualisiert werden, sondern verweisen stets auf komplex vermittelte intermediäre Risikozustände, die schließlich in manifeste Störungsbilder einmünden können [93, 168].

**Zusammenfassend** betonen epidemiologische Studien einhellig, dass eine Koexistenz/Komorbidität von PTBS und Substanzkonsumstörung mit einem schwerwiegenderen klinischen Symptombild in akuten Krankheitsepisoden, mit häufigeren stationären Notfallaufnahmen, reduzierten Ansprechraten auf durchgeführte Behandlungen, einem schwierigeren und komplikationsreicheren Krankheitsverlauf, mit höheren interpersonalen Problemen, massiveren psychosozialen Behinderungsgraden und niedrigerer Gesundheits-bezogener Lebensqualität einhergeht. Eine bedeutsamere Suizidalität und ein erhöhtes Gesamtmortalitätsrisiko sind besonders zu beachten [63, 121, 138].

## Substanzkonsumstörung als Risikofaktor für Trauma und PTBS

Eine von Substanzabusus bestimmte Lebenspraxis birgt per se ein erhöhtes Risiko für eine Reihe von möglichen Trauma-Expositionen. In diesem Fokus darf ebenfalls keine lineare Kausalversursachung aus der zeitlichen Abfolge von Substanzabusus, Traumaerfahrung und PTBS abgeleitet werden. In einer biopsychosozialen Perspektive ist zunächst grundlegend, dass **soziale Kontexte**, in denen Personen aufwachsen und leben, sowohl eine Reihe von gebündelten Traumata und chronischen Stressoren mit erhöhten Risiken für eine PTBS als auch gleichzeitig für Substanzkonsumstörungen vermitteln können [35, 82, 173]. In einem Substanzabusus *und* Traumatisierung bahnenden „Envirom“ üben die **allgemeinen sozialen Determinanten von Gesundheit und Krankheit** (sozialer Gradient, Stressoren/Traumata, frühe Entwicklung und Beziehungssicherheit, Erziehungs- und Ausbildungsqualität, soziale Exklusion, Diskrimination und Armut, Kontrolle über Arbeit, Jobsicherheit und Arbeitslosigkeit, soziale Unterstützung und Zugang zum Gesundheitssystem, Gebrauch von Sucht-stiftenden Substanzen, Qualität von Ernährung und Essverhalten) einen grundlegenden und anhaltenden Einfluss aus [120]. Hierbei ist nicht nur ein **„Zuviel“ an Risikofaktoren**, sondern in der Regel auch ein **„Zuwenig“ an protektiven Faktoren** bedeutsam [183]. Einige **spezielle Aspekte** eines krisenhaft entgleitenden oder chronischen Substanzkonsums, die inhärent eine vermehrte Exposition gegenüber Traumata und damit assoziiert ein erhöhtes PTBS-Risiko vermitteln, können zudem aufgezeigt werden:

- Hohe Raten an interpersonaler Gewalt, sexueller Ausbeutung und lebensgefährlichen Verletzungen charakterisieren eine **traumatisierende soziale Lebenswelt** zahlreicher drogenabhängiger Personen in ihrer alltäglichen Beschaffung von Substanzen [75, 86, 87, 169].
- Substanzmissbrauch und interpersonale Gewalterfahrungen definieren ein hoch komplexes Risikobündel speziell für **Substanz-abhängige Frauen** [3, 36].
- Der Zusammenhang von Substanzabusus (z. B. Rauschtrinken, „binge drinking“, Erstkonsum von illegalen Drogen) und erhöhtes Traumaexpositionsrisiko stellt in **Adoleszenz und jungem Erwachsenenalter **eine besondere entwicklungspsychopathologische Herausforderung dar [49, 60, 110, 144].
- Alkohol- und Drogen-induzierte **Intoxikationen** (v. a. beinah-letale Überdosierungen von Opiaten) implizieren unter speziellen körperlichen und psychosozialen Bedingungen ein hohes Risiko an gefährlichen Verletzungen, traumatisch erlebten Reanimationen, invasiv-chirurgischen und intensivmedizinischen Behandlungen. Sie alle können peritraumatische Symptome sowie in Folge auch eine posttraumatische Verarbeitung anstoßen [9, 37, 40, 88, 116].

**Zusammenfassend** sind für die Relation von Substanzkonsumstörung und Trauma/PTBS empirisch zahlreiche intermittierende Variablen zu beachten, die ein komplex interagierendes Bedingungsgefüge aufspannen. Substanzabusus im Kontext von Armut, Viktimisierung, sozial devianten Lebensverhältnissen führt zu kumulativen Risiken für Trauma und PTBS, die wiederum im Sinne einer eskalierenden Spirale den Substanzkonsum verstärken. Einige spezielle traumatogene Aspekte, die einem Leben mit chronischem Substanzkonsum oft inhärent sind, müssen hierbei beachtet werden. Zudem sind differentielle pharmakologische Effekte eines Substanzkonsums zu diskutieren, die auf neurobiologischer Ebene auch ein erhöhtes PTBS-Risiko nach einer Traumatisierung beinhalten (siehe unten).

## Trauma/PTBS als Risikofaktor für Substanzkonsumstörung – Selbstmedikation bei PTBS-Symptomen

Eine klinisch attraktive Hypothese (**Selbstmedikation**) besagt, dass Personen mit schwerwiegenden psychischen Störungen, wie beispielsweise PTBS bestimmte psychotrope Substanzen mit dem subjektiven Ziel vermehrt einsetzen, um definierte PTBS-Symptome und einen hiermit assoziierten emotionalen Distress zu lindern oder aus dem PTBS-Verlauf resultierende psychosoziale Stressoren besser zu kupieren. Ein verstärkter Substanzkonsum wird in dieser Sichtweise als eine Variante der Selbstmedikation in Ermangelung einer effizienteren Coping-Strategie verstanden [97].

Empirische Belege für diese Hypothese stammen zunächst aus zahlreichen **epidemiologischen** Studien, die nach schwerwiegenden Traumatisierungen eine nahezu parallele Entwicklung zwischen der Intensität der PTBS-Symptome und dem Ausmaß des jeweils unterhaltenen Substanzkonsums (Alkohol, Nikotin, Benzodiazepine, Opiate, Cannabis) im weiteren Verlauf abbildeten [26, 29, 56, 70, 78, 206]. Ergebnisse aus **Langzeitstudien**, die eine häufigere inzidente Substanzkonsumstörung nach Trauma und PTBS, nicht aber nach Trauma-Exposition alleine hervorhoben, gehen mit dieser Sichtweise ebenfalls gut konkordant [31, 158]. Belege für eine Selbstmedikationshypothese erbrachten auch Detailstudien, die einen **erhöhten Substanzkonsum **in Abhängigkeit von definierten **PTBS-Symptomclustern **(Hyperarousal, Vermeidung, intrusive Wiedererinnerung; negative Trauma-assoziierte Stimmungen und Kognitionen) als Strategien einer Selbstregulation analysierten [29, 57, 62, 80, 124, 172, 201]. Generell stellte sich ein signifikanter Zusammenhang zwischen dem Gesamt-Score über alle PTBS-Symptomcluster und der Intensität des berichteten **Cravings** für die untersuchten Substanzen (Alkohol, Stimulantien, Opiate) dar. Es zeichneten sich bei bevorzugt eingesetzten Substanzen **(„drug of choice“**) einige typische Assoziationen zu speziellen PTBS-Symptomclustern ab (Stimulantien: PTBS-Vermeidung; Alkohol: PTBS-Hyperarousal; Opiate: kein spezielles PTBS-Symptommuster [179]).

Eine Reihe von Studien konzentrierte sich auf **Variablen**, die den Zusammenhang von PTBS und koexistenter Substanzkonsumstörung vermitteln könnten. Personen mit PTBS setzen offenkundig Substanzen vor allem in Augenblicken heftiger, nur schwer kontrollierbarer Emotionen ein, um hierüber zu einer günstigeren Selbstkontrolle zu gelangen [28, 191, 192]. Im Kontext einer PTBS bezieht sich eine **emotionale Regulationsstörung** nicht nur auf massive **negative **Affektzustände. Sie kann auch bei starken **positiven** Emotionen aufgezeigt werden [204, 205]. Als wahrscheinlicher Pfad zu einem verstärkten koexistenten Substanzkonsum zeichnet sich ab: Heftige emotionale Zustände mit Bezug zu Traumaerinnerung und -verarbeitung überfordern die Regulationsfähigkeit einer Person. Sie gehen mit einer reduzierten Selbstwirksamkeit einher. Sie führen zu einem erhöhten subjektiven Distress und triggern eine Reihe von potenziell gesundheitsschädigenden Verhaltensweisen (z. B. riskantes Sexual-, aggressives Verhalten). Sie favorisieren auch den Einsatz einer psychotropen Selbstmedikation, die als Risikoverhalten für eine nachfolgende Substanzkonsumstörung gewertet werden muss [1, 155, 157, 181, 203]. Das Persönlichkeitsmerkmal **Impulsivität** wird über die unmittelbar durch einen Trauma-Triggerreiz ausgelöste emotionale Antwort angezeigt. Impulsivität vermittelt als Co-Risikofaktor die Assoziation von PTBS-Symptomintensität und **koexistentem Alkoholkonsum** bedeutsam [119, 160]. Dieser Zusammenhang kann für andere Drogen aber nicht in vergleichbarer Weise bestätigt werden. Bei **koexistenter Kokainabhängigkeit** etwa wird die Aufrechterhaltung eines Konsums sehr viel stärker von einer sich bietenden situativen Gelegenheit relativ unabhängig vom PTBS-Status gesteuert [190, 198]. Die Relation von PTBS und koexistenter Alkoholkonsumstörung nach **sexueller Traumatisierung** wird insbesondere bei Frauen stark durch die kognitive Variable einer Selbstschuld-Attribution (**„self-blaming“**) beeinflusst [99, 147]. Bei einer Vergewaltigung unter Alkoholeinfluss und nachfolgender PTBS sind **zustandsabhängige intrusive Trauma-Erinnerungen** unter erneutem Alkoholkonsum und eine zusätzlich verstärkte PTBS-Symptomatik in Folge zu bedenken [83].

**Zusammenfassend **lässt sich die epidemiologisch hohe Koexistenz/Komorbidität von PTBS und Substanzkonsumstörung teilweise auch als Folge eines vorrangig emotionsgesteuerten Copings bei PTBS verstehen, als Versuch mittels psychotroper Substanzen eine günstigere Selbst- und Emotionskontrolle zu erreichen.

## Neurobiologische Konsequenzen von Substanzmissbrauch und -abhängigkeit in Konvergenz mit der Pathophysiologie der PTBS

Alle psychotropen Substanzen, die ein Suchtpotential aufweisen, beeinflussen das **natürliche Belohnungssystem** entweder direkt oder indirekt durch einen vermehrten **Dopamin**-Turnover speziell in den limbischen Bahnen zwischen der Area tegmentalis ventralis des Mittelhirns und dem Nucleus accumbens (ventrales Striatum) der Basalganglien [135]. Das Ausmaß der dopaminergen Aktivierung an den DA_2_-Rezeptoren des Ncl. accumbens ist unmittelbar mit der Raschheit und Intensität der ausgelösten Belohnung korreliert. Das subjektive Genusserlebnis selbst wird aber nicht durch Dopamin, sondern vorrangig durch endogene **Opioide, Endocannabinoide** und** GABA **vermittelt [106]. Die Belohnungsvalenz einer bestimmten Substanz und des hierauf gerichteten Konsumverhaltens wird unter Einfluss von Amygdala und Hippokampus auch an den speziellen situativen Kontext des Konsums gebunden. Diese Lernschritte zur Motivation und Realisierung eines bestimmten Konsumverhaltens werden unter kognitiver, evaluativer und exekutiver Beteiligung des präfrontalen und motorischen Kortex wesentlich über Glutamat reguliert [90, 91]. Ein zentrales neurobiologisches Korrelat in der Entwicklung einer Substanzkonsumstörung stellt die molekulare **Gegenregulation im Belohnungssystem** selbst dar. Diese vollzieht sich vorrangig über eine verstärkte Aktivierung der κ‑Opiatrezeptoren durch **Dynorphin.** Sie führt zu einer Verminderung der belohnenden und analgetischen Effekte von endogenen Opioiden/zugeführten Opiaten an den μ‑ und δ‑Opiatrezeptoren (*Toleranzentwicklung*). Andererseits kommt es über eine parallele **Aktivierung der evolutionären Stresssysteme** (HPA-Achse, autonomes Nervensystem, Immun‑/Inflammationssystem; **CRF, Kortisol, Nordrenalin/Adrenalin, Zytokine**) zu einer weiteren Gegenregulierung. Diese Systeme induzieren eine erhöhte Stressanfälligkeit (*Entzugssymptomatik*) und intensivieren gleichzeitig ein verstärktes Suchverhalten nach belohnenden Substanzen. Das Grundmerkmal einer jeden Sucht, nämlich eine immer stärker eskalierende Diskrepanz zwischen einem sensitivierten Craving („*wanting*“) einerseits und einem reduzierten Belohnungsempfinden („*liking*“) andererseits, geht mit einer Umstellung der Verhaltensinitiierung von einem Modus der „*positiven Verstärkung*“ auf einen der „*negativen Verstärkung*“ einher [16, 17, 105]. Die Verhaltensausführung vollzieht sich hierbei unter einer bedeutsam eingeschränkten *regulativen Funktionalität* präfrontaler Strukturen [111].

In einer **neurobiologischen **Sicht auf die Koexistenz/Komorbidität von **PTBS und Sucht** ist grundlegend, dass beide Störungen prinzipiell innerhalb derselben neuronalen Regulationssysteme organisiert werden und gemeinsame molekulare Mechanismen teilen. Sie können sich in ihrem Verlauf wechselseitig verstärken [52, 53, 121, 184].

- **Akuter Stress** (z. B. bei Trauma-Exposition) bewirkt zunächst ganz ähnlich wie Substanzen mit suchtstiftendem Potenzial eine starke dopaminerge Aktivierung der Dopaminrezeptoren im Ncl. accumbens. Stress löst in weiterer Folge auch die skizzierte Kaskade einer vermehrten Freisetzung von endogenen Opioiden, Endocannabinoiden und GABA, aber auch von Glutamat aus. Hieran sind auch eine aktivierte HPA-Achse sowie ein erhöhter noradrenerger Turnover beteiligt.
- **Chronischer Stress,** wie paradigmatisch eine **PTBS**, mündet in einen vergleichbaren Reaktionszustand des Belohnungssystems ein, wie er für einen chronischen Alkohol- oder Drogenkonsum charakteristisch ist. Auch eine PTBS geht mit einem auffälligen Belohnungsdefizit im Nucleus accumbens einher [134]. Diese depressions-ähnliche Anhedonie wird nach DSM‑5 im D‑Cluster einer PTBS kodiert. Eine PTBS kann, ganz analog wie Substanzkonsumstörungen, auch mit prominenten kognitiven, evaluativen und exekutiven Funktionsdefiziten im präfrontalen Kortex assoziiert sein [38].
- Die **sensitivierten Stresssysteme **bei **PTBS** (v. a. sensitiviertes Noradrenalinsystem, dysfunktionale HPA-Achse) nehmen einen signifikanten Einfluss auf die Aufnahme und Intensivierung von suchtfördernden Substanzen. Sie erhöhen die Rückfallgefährdung nach intermittierend erreichter Abstinenz. Akute und chronische PTBS-Stressoren verstärken die Effekte von Substanz-bezogenen Schlüsselreizen auf das Dopaminsystem. Unter den Bedingungen der langfristigen neuroadaptiven Veränderungen des Belohnungssystems und der hiermit vernetzten kortikalen Kontrollstrukturen wird das inhärente Dilemma einer koexistenten Substanzkonsumstörung noch weiter eskaliert: Eine drang- und letztlich zwangsbestimmte Verhaltensregulation in der Beschaffung psychotroper Substanzen („*wanting*“; Glutamat, DA_1_-Rezeptoren) wird gebahnt. Gleichzeitig gelingt die Realisierung des angestrebten Genusseffektes („*liking*“; reduzierte Ansprechbarkeit der DA_2_-Rezeptoren) immer weniger. Substanzen dienen vorrangig der vorübergehenden Beruhigung des induzierten emotionalen Distresses und der korrelierten physiologischen Stressreaktionen. Die Sucht-inhärente Stressaktivierung wirkt sich wiederum nachteilig intensivierend auf einzelne PTBS-Cluster (v. a. intrusive Traumaerinnerung, autonomes Hyperarousal) aus [54, 197].

**Einzelne Substanzen mit Suchtpotential** besitzen neben dem **allgemeinen pharmakologischen Wirkprofil** auf das Belohnungssystem auch einige **differentielle Effekte**, die im Kontext der Pathophysiologie der PTBS Relevanz besitzen:

- **Alkohol** wird von PTBS-Patient*innen in seinem stark Distress-reduzierenden Effekt geschätzt und häufig für ein günstigeres Selbstmanagement eingesetzt. Alkohol beruhigt, entspannt, entängstigt und wirkt in steigenden Dosen Schlaf-anstoßend. Seine pharmakologische Hauptwirkung wird über den GABA_A_-Rezeptor vermittelt, der zu einer Verschiebung in der Balance zwischen hemmendem GABA- und exzitatorischem Glutamat-System führt. Nach einer Trauma-Exposition zeichnen sich bei einem Übergang in eine PTBS stark reduzierte kortikale GABA-Spiegel (v. a. parietal, okzipital, anteriores Zingulum, Insel) bei gleichzeitig erhöhten Glutamat-Konzentrationen (v. a. temporal) ab. Die Schwere der PTBS-Symptomatik, insbesondere der Schlafstörungen ist hiermit korreliert [127, 162]. Alkoholkonsum kann dieses GABA-erge Defizit bei PTBS-Patienten zumindest vorübergehend mindern und auch die Intensität von aktuellen PTBS-Symptomen abschwächen [145]. Alkohol mitigiert zwar eine Stress-induzierte Erhöhung des Cortisol- und Noradrenalin-Outputs, chronischer Alkoholkonsum oder starkes Rauschtrinken („binge drinking“) sind aber mit erhöhten tonischen Kortisol- und Noradrenalinspiegeln assoziiert [207]. Chronischer Alkoholkonsum verringert zudem die GABA-Rezeptoren und erhöht kompensatorisch die glutamatergen NMDA-Rezeptoren. Beide neurobiologische Anpassungen tragen zu einer Stress-Sensitivierung bei, die sowohl die Alkoholkonsumstörung als auch die PTBS intensivieren und komplizieren [5, 76].
- **Nikotin **wird ähnlich häufig wie Alkohol von PTBS-Patient*innen als Selbstmedikation im Umgang mit den posttraumatischen Symptomen verwendet [95]. Nikotin zeichnet sich durch ein polyvalentes Wirkprofil aus. Es wirkt gleichzeitig sedierend, anxiolytisch, antidepressiv, konzentrationsfördernd und allgemein stimulierend. Es entfaltet seine Wirkung über die präsynaptischen nikotinergen Acetylcholinrezeptoren, v. a. vom α_4_β_2_-Subtyp. Es kommt dosis- und kontextabhängig hierüber zu einer erhöhten zerebralen Verfügbarkeit von Dopamin, Serotonin, β‑Endorphinen, Noradrenalin, Acetylcholin und Vasopressin [10]. Bei chronischem Nikotinkonsum überwiegen aber die Stress-induzierenden Effekte einer aktivierten HPA-Achse mit vermehrter Sekretion von Kortisol und eines sensitivierten Noradrenalinsystems mit je nachteiligen Effekten sowohl auf den Verlauf der PTBS als auch der koexistenten Nikotin-Konsumstörung [207].
- **Benzodiazepine** zeigen mehrfache tranquilisierende Effekte (anxiolytisch, sedierend, schlafanstoßend, muskelrelaxierend, antiepileptisch). Sie entfalten eine agonistische Wirkung am GABA_A_-Rezeptor des hemmenden GABA-Systems. Tierexperimentell kann über GABAerge Interneurone ein modulierend hemmender Effekt auf die Konsolidierung von aversiven/traumatischen Erinnerungen in den basolateralen Anteilen der Amygdala nachgewiesen werden [118, 161]. Es ließe sich hieraus ableiten, dass nach einer Traumaexposition der **prophylaktische Früheinsatz **von Benzodiazepinen einer noradrenergen, aber auch glutamatergen Hyperaktivität entgegenwirken und eine überstarke Konsolidierung verhindern könnte. Die wenigen empirischen psychopharmakologischen Studien an Patientensamples bestätigten diese Hypothese aber nicht. Benzodiazepine waren im Vergleich zu Placebo nicht nur nicht überlegen, sie erhöhten nach einem Trauma sogar die Übergangswahrscheinlichkeit zu PTBS und Depression [65, 126]. Diese im klinischen Kontext potenziell nachteiligen Effekte von Benzodiazepinen im akuten Routineeinsatz nach einem Trauma werden mit einer dysmnestischen und dissoziativen Wirkung auf die Organisation des Trauma-Gedächtnisses und mit einer Behinderung des Extinktionslernens in Verbindung gebracht. Benzodiazepine zeigen ferner einen hemmenden Effekt auf die HPA-Achse und reduzieren die Sekretion von Kortisol [215]. Niedriges Kortisol wird aber als Risikofaktor für PTBS nach einem Trauma diskutiert [212]. Eine beeinträchtigte hemmende Funktion auf die intrusiven Traumaerinnerungen bei erniedrigtem Kortisol wird postuliert [154]. Benzodiazepine können die Symptomcluster bei einer etablierten **PTBS** nicht signifikant bessern [13]. Trotzdem sind die Verordnungszahlen/Konsumraten von Benzodiazepinen bei PTBS-Patient*innen, vor allem bei Frauen nach wie vor sehr hoch [13, 15, 100]. Die nachteiligen Effekte eines chronischen Benzodiazepinkonsums sind in ihren neurobiologischen Auswirkungen auf die PTBS-Pathophysiologie ähnlich denen eines chronischen Alkoholkonsums zu bewerten (siehe oben).
- **Cannabinoide **wie **Cannabis** (psychoaktive Komponente: ∆_1_-Tetrahydrocannabinol, THC) oder **Cannabidiol** (CBD) entfalten ihre Effekte über die CB_1_- und CB_2_-Rezeptoren auf das Endocannabinoidsystem des Gehirns. THC wirkt an den CB_1_- und CB_2_-Rezeptoren partiell agonistisch. CBD hingegen ist am CB_1_-Rezeptor antagonistisch und kann einen Teil der THC-Effekte aufheben. Cannabinoiden werden zahlreiche Effekte zugeordnet, von denen die Wirkungen bei chronischen Schmerzsyndromen, Übelkeit und Erbrechen bei Chemotherapie, Spastizität bei Multipler Sklerose noch die gesichertste empirische Evidenz besitzen [133]. Cannabisprodukte werden wegen ihrer emotional ausgleichenden Wirkung im Vergleich zur Allgemeinbevölkerung dreimal häufiger von PTBS-Patient*innen konsumiert [96]. Das hierüber aktivierte Endocannabinoidsystem nimmt einen modulierenden Einfluss auf die Konsolidierung des Gedächtnisses, den Retrieval-Prozess und die Extinktion von emotionalen und traumatischen Erfahrungen in den Kernregionen der basolateralen Amygdala, des Hippocampus und des präfrontalen Kortex. CB_1_-agonistische Cannabinoide wie z. B. THC stören den Retrievalprozess (Blockade der unwillkürlichen Traumaerinnerungen), fördern aber möglicherweise das Extinktionslernen [142]. Bei PTBS-Patient*innen waren die Konzentrationen des Endocannabinoids Anandamid erniedrigt, die Verfügbarkeit des CB_1_-Rezeptors kompensatorisch erhöht. Die CB_1_-Rezeptorverfügbarkeit in der Amygdala korrelierte positiv mit einer abnormen Prozessierung von Gefahrenreizen und einem erhöhten Niveau eines autonomen Arousal, aber nicht mit dysphorischen Symptomen [136]. Cannabiskonsum kann einige PTBS-Symptome abschwächen. Für das synthetische Cannabinoid Nabilone wurde in einem randomisierten, doppelblinden und placebokontrollierten Cross-over-Design eine gute Wirksamkeit bei PTBS-assoziierten Albträumen nachgewiesen [85]. Selektive Cannabinoide werden in der Behandlung der PTBS nach positiven Einzelfallstudien unter Beachtung der vorliegenden neurobiologischen Befunde zum Endocannabinoidsystem als eine weitere pharmakologische Therapievariante diskutiert [77]. Rezente systematische Reviews mit Metaanalyse hielten für den therapeutischen Einsatz von Cannabinoiden bei PTBS derzeit aber eine nur eine sehr geringe Evidenz für positive Effekte auf die PTBS-Symptomatik fest [19, 140]. Eine koexistente Cannabiskonsumstörung kann zudem zahlreiche PTBS-assoziierte Probleme wie kognitive und motivationale Defizite weiter verschärfen. Langfristig persistierende maladaptive Veränderungen in der HPA-Achse und im Noradrenalinsystem sind im Hinblick auf den PTBS-Verlauf nachteilig zu bewerten [207]. Die problematischen Effekte von Cannabis auf die Reifungs-abhängige zerebrale Reorganisation in Adoleszenz und jungem Erwachsenenalter sind besonders kritisch zu bedenken [72].
- **Amphetaminerge Stimulantien** und **Kokain** werden von einer Subgruppe von PTBS-Patient*innen ebenfalls überproportional häufig konsumiert [68, 164, 188]. Die euphorisierenden und allgemein aktivierenden Effekte werden über komplexe Mechanismen vermittelt. Ein pharmakologischer Hauptpfad hebt hervor: Kokain und Amphetamine sind kompetitive Substrate für Dopamin‑, Noradrenalin- und Serotonin-Transporterproteine. Stimulantien gelangen statt der monoaminergen Neurotransmitter, die so vermehrt im synaptischen Spalt verbleiben, in das präsynaptische Terminal. Hier blockieren sie die Aufnahme der lokal synthetisierten Monoamine in die präsynaptischen Vesikeln durch eine Blockade der vesikulären Monoamintransporter 2 (VMAT_2_) und führen zu einer verstärkten Freisetzung der Monoamine. Eine Blockade der Monoaminoxidasen bewirkt eine zusätzliche Anreicherung von Dopamin, Noradrenalin und Serotonin im synaptischen Spalt [45, 123]. Amphetamine und Kokain können das PTBS-assoziierte Belohnungsdefizit vorübergehend wirksam antagonisieren, münden aber über den allgemeinen Sucht-stiftenden Circulus vitiosus sekundär in dieses ein und verstärken es signifikant [4]. Eine koexistente Stimulantien-Konsumstörung geht mit einer Intensivierung des Hyperarousal-Clusters einer PTBS einher und fördert wiederum einen verstärkten missbräuchlichen Beikonsum von tranquilisierenden Substanzen wie Alkohol, Benzodiazepinen, Opiaten oder Cannabis [51, 208]. Bedeutsame kognitive Defizite, aber auch erhöhte andere neuropsychiatrische, kardio- und zerebrovaskuläre Risiken sind bei einer Koexistenz mit PTBS zusätzlich zu berücksichtigen [67, 153].
- Das **3,4-Methylendioxy-N-Methylamphetamin** (**MDMA, Ecstasy**) zeigt eine stimmungsaufhellende, Empathie und sozialen Kontakt fördernde Wirkung. MDMA erfreut sich einer großen Beliebtheit in der Partyszene. Die **missbräuchliche Einnahme** von **MDMA **zeigt ähnlich bedeutsame neuropsychiatrische Nebenwirkungen wie auch andere Stimulantien [194]. Für den PTBS-Verlauf ist eine koexistente MDMA-Konsumstörung vergleichbar problematisch einzustufen. Der pharmakologische Hauptwirkmechanismus von MDMA ist den klassischen Amphetaminen sehr ähnlich, die im ZNS verstärkte Freisetzung von Dopamin ist im Vergleich zu Noradrenalin und Serotonin aber geringer. Weitere neurobiologische Effekte sind eine Reduktion der amygdalären Hyperaktivität bei gleichzeitiger Aktivierung präfrontal-kortikaler Strukturen, eine Erhöhung der Oxytocin-Spiegel, eine positive Beeinflussung von Furchtextinktion und Gedächtniskonsolidierung. Diese speziellen Effekte werden derzeit hinsichtlich eines möglichen therapeutischen Potentials bei der PTBS diskutiert [108]. Erste kontrollierte Studien zur **Augmentation von Trauma-Psychotherapien** (z. B. prolongierte Exposition) **mittels MDMA** haben zu ermutigenden Ergebnissen geführt [7, 59, 84]. Angesichts noch nicht klar definierter Ausschlusskriterien, einer noch ungenügend erprobten Dosisfindung und weitgehend fehlender Erfahrungen in der Langzeitanwendung wird eine vorsichtige Zurückhaltung empfohlen [165]. Die vorliegende empirische Datenlage zur Wirksamkeit und Sicherheit von MDMA zur Augmentation von Trauma-Psychotherapien wird für eine FDA-Zulassung derzeit als noch nicht ausreichend beurteilt [159].
- **Opiate** werden gleichfalls von zahlreichen PTBS-Patient*innen, speziell mit assoziierten chronischen Schmerzsymptomen zur Selbstmedikation eingesetzt [143]. Zwischen einer **Opiat-Konsumstörung**, einem **chronischen Schmerzsyndrom** und einer **PTBS** bestehen große neurobiologische Parallelen. Alle drei klinischen Syndrome konvergieren in einem auffälligen **„Belohnungsdefizit“**, das sich durch eine verringerte Aktivierung der dopaminergen DA_2/3_-Rezeptoren im Ncl. accumbens (ventrales Striatum) darstellen lässt [25, 52, 53].
- Die Zusammenhänge von Trauma, PTBS und endogenes Opiatsystem sind komplex. Wichtige klinische Argumente für eine Beteiligung des Opiatsystems an der Pathophysiologie der PTBS sind [92]: Vor allem Frauen mit PTBS nach interpersonellen Gewalttraumata zeigen eine hohe Rate an chronischen Schmerzsyndromen. Personen mit sexuellem Missbrauch in ihrer frühen Entwicklung weisen eine signifikant erhöhte Rate an Opiatsubstanzmissbrauch auf. Eine der bedeutsamsten prophylaktischen pharmakologischen Maßnahmen nach schweren traumatischen Verletzungen und heftigen akuten Schmerzzuständen ist hinsichtlich eines späteren PTBS-Risikos die Gabe hoher Dosen von Morphinen. Entzugssymptome können nach einer prolongierten Stresssituation auftreten oder aber durch die Gabe des Opiatantagonisten (z. B. Naloxon) ausgelöst werden. Bei PTBS-Patient*innen finden sich im Liquor erhöhte Konzentrationen an β‑Endorphinen.
- Endogene Opioide entfalten über ein heterogenes Muster an μ‑, δ‑, k‑Opiatrezeptoren differenzielle Wirkungen [193]. Sie hemmen bei Trauma-Exposition die **Schmerzwahrnehmung** und reduzieren die v. a. noradrenerg getriggerten **Panikaffekte**. Die Amygdala ist besonders reich an Opiatrezeptoren. Eine über Opioide vermittelte **psychomotorische Erstarrung** und **affektive Betäubung** („freezing/numbing“) erlaubt möglicherweise dem Organismus, einen überwältigenden Stress nicht bei klarem Bewusstsein zu überstehen und auch die traumatische Erfahrung nicht exakt zu speichern. Eine negative Interferenz mit Lern- und Gedächtnisprozessen ist die Folge hoher endogener Opiatkonzentrationen. Ein differenzielles μ‑Opiatrezeptorbindungsverhalten stellte sich in einer PET-Studie bei Personen nach einer Traumaexposition mit und ohne nachfolgende PTBS dar. Beide traumaexponierten Gruppen unterschieden sich von gesunden Kontrollprobanden ohne Traumaerfahrung durch eine niedrigere Bindungskapazität in der erweiterten Amygdala, im Nucleus accumbens sowie im frontalen und dorsalen insulären Kortex und durch eine höhere Bindung im orbitofrontalen Kortex. PTBS-Patient*innen wiesen niedrigere Werte im anterioren Zingulum auf gegenüber den beiden anderen Gruppen, traumaexponierte Personen ohne PTBS wiederum niedrigere Werte in der Amygdala, aber höhere Werte im orbitofrontalen Kortex (OFC) im Vergleich zu den beiden anderen Gruppen [117]. In einer weiteren PET-Studie, an der in einem transdiagnostischen Ansatz nicht nur PTBS-Patient*innen, sondern auch Patient*innen mit generalisierter Angststörung und Major Depression teilnahmen, war die Verfügbarkeit von κ‑Opiatrezeptoren mit dem Dysphoriesymptomcluster korreliert [150].
- Die kurzfristigen adaptiven Vorteile der opioidvermittelten Stressreaktionen müssen gegen die **hinderlichen Langzeiteffekte** einer prolongierten **Dissoziation** aufgerechnet werden. Der zustandsabhängige erhöhte Opiatgehalt während einer Traumaexposition ist möglicherweise in einen pathogenetischen Kontext zu stellen, wenn Personen mit PTBS im weiteren Verlauf eine Tendenz zeigen, nicht nur verstärkt Opiate zu konsumieren, sondern auch Situationen mit einem hohen Retraumatisierungsrisiko aufzusuchen („**trauma addiction**“) [103, 104, 167]. Diese auffällige Verhaltenstendenz ist möglicherweise bei jenen PTBS-Personen mit hohem Score für „**riskante Verhaltensweisen**“ (Cluster D) besonders ausgeprägt [178]. In Kasuistiken wird in diesem Zusammenhang speziell über Soldaten berichtet, die schwer traumatisiert aus einem Kampfeinsatz zurückkehren und sich trotz ausgeprägter PTBS-Symptomatik erneut für ein hoch riskantes Kriegsengagement melden. Sie schildern nicht selten einen „Rausch“, den sie während eines aktiven Gefechts erleben [33, 132, 177]. Für den Verlauf von PTBS und koexistenter Opiat-Konsumstörung ist ein sich wechselseitig verstärkender neurobiologischer Ciculus vitiosus charakteristisch [14, 42, 58].

**Zusammenfassend **ist für das Verständnis der Koexistenz/Komorbidität von PTBS und Substanzkonsumstörung in neurobiologischer Sicht **allgemein** festzuhalten, dass beide Störungen innerhalb gemeinsam neuronaler Strukturen und Mechanismen interagieren, wobei ein vermindertes Belohnungsempfinden und eine sensitivierte Stressreagibilität als klinische Merkmale hervorstechen. **Spezifischen** Effekten einzelner Substanzen auf die PTBS und deren Verlauf ist in dieser allgemeinen neurobiologischen Dynamik differentiell Rechnung zu tragen.

Die bisherige Diskussion der Koexistenz/Komorbidität von PTBS und Substanzkonsumstörung galt der Untersuchung in einer bidirektionalen Wirkrichtung, mit einem Blick auf Substanzkonsumstörung als Risikofaktor für Trauma und PTBS zum einen, auf Trauma und PTBS als Risikofaktor für Substanzkonsumstörung zum anderen. Sie betonte ferner auffällige Konvergenzen in den neurobiologischen Prozessen beider Störungen. Es ist aber auch ein Analysefokus möglich, der auf Faktoren gerichtet ist, die möglicherweise beiden Störungen gemeinsam zugrunde liegen. Hierbei sind vor allem genetische Aspekte, bestimmte Persönlichkeitsdimensionen und spezielle Aspekte der frühen Entwicklung näher ins Auge zu fassen.

## Genetische und epigenetische Faktoren als gemeinsame Basis von PTBS und Substanzkonsumstörungen

Sowohl die PTBS als auch Substanzkonsumstörungen (allgemein, speziell) zeichnet eine vielfältige genetische Basis aus, die je für sich eine besondere Vulnerabilität versus Resilienz unter definierten Umwelteinflüssen epigenetisch mitbestimmt (**PTBS**: [48, 81, 122, 137, 170]; **Substanzkonsumstörungen allgemein**: [11, 23, 64, 71, 189]; **Alkohol:** [43] **Nikotin**: [148]; **Cannabis**: [131]; **Psychostimulantien**: [89, 149]); **Opiate**: [24, 202]). Die genetische Forschung hat bisher zur Koexistenz/Komorbidität von PTBS und definierten Substanzkonsumstörungen erst einige wenige Studien vorgelegt:

- Für die **Koexistenz** von **PTBS und Alkoholabhängigkeit** konnte in **Zwillingsstudien** eine hohe genetische Überlappung nachgewiesen werden. An einem großen Sample weiblicher Zwillinge (PTBS + Alkoholmissbrauch; *N* = 3768; Alter: 18–29 Jahre) erklärten additive Alkoholismus-assoziierte genetische Einflüsse über 70 % der PTBS-Varianz. Die verbleibende PTBS-Varianz war auf Individuen-spezifische und nicht auf gemeinsam geteilte Umweltfaktoren zurückzuführen. Die genetische Korrelation zwischen PTBS und Alkoholkonsumstörung betrug 0,54 [163]. Sie lag bei diesem Sample von Frauen höher als bei früheren Untersuchungen an männlichen Zwillingen (PTBS + Alkohol‑, Cannabismissbrauch) aus dem Vietnam Era Twin Registry [101, 125, 210, 211]. Für ein **männliches Zwillings-Sample** (Vietnam Era Twin Registry) stellte sich eine ähnliche genetische Überlappung für die Koexistenz von **PTBS** und **Nikotinabhängigkeit** dar [102].
- Mehrere **molekulargenetische** Studien konzentrierten sich auf den möglichen Einfluss spezieller genetischer Varianten. Die Relevanz von Genpolymorphismen für die **α**_**2**_**-Untereinheit des GABA-Rezeptors** (**GABA α**_**2**_) wurde sowohl für die **PTBS** als auch für den missbräuchlichen Konsum von **Alkohol, Kokain** und **Heroin** nachgewiesen [55]. Polymorphismen des **GABA-Transporter-Gens**_**1**_ (**GAT1**), das in einer engen Verbindung mit der Enkodierung von emotionalen und Angsterinnerungen steht, wurden als signifikant sowohl für die Assoziation **PTBS-Depression** als auch für die Assoziation **PTBS-Substanzmissbrauch** erkannt [27]. Ein **α‑Synuclein** Polymorphismus (**SNCA rs356195**) vermittelte den Zusammenhang zwischen PTBS-Symptomatik und **riskantem Alkoholkonsum**, war aber nicht mit Aggressionsmaßen eines externalisierenden Verhaltens assoziiert [69]. Ein Polymorphismus für den **Dopamin**_**2**_**-Rezeptor **(**TaqI A1 Allel**) konnte in seinem Einfluss auf PTBS-Patienten mit **starkem Alkoholkonsum** identifiziert werden [213]. Dieser Polymorphismus war auch mit einem ausgeprägten **Belohnungsdefizit** [22] und tierexperimentell mit dem Merkmal **Impulsivität** zu verbinden [41]. Der für die allgemeine Stressregulation bedeutsame **5‑HTTLPR** Polymorphismus des **Serotonintransporters** scheint bei PTBS auch mit einem **verstärkten Alkoholkonsum** gekoppelt zu sein [94]. Für die Assoziation von **PTBS** und **Nikotinabhängigkeit **wurden **Genvarianten CHRNA5-A3-B4** des **nikotinergen Acetylcholin-Rezeptors** diskutiert [34].
- Eine Reihe von gemeinsam (>1 %) vorkommenden **Einzel-Nukleotidpolymorphismen** (**SNPs**) üben ebenfalls moderate genetische Einflüsse sowohl auf die PTBS-Symptomatik nach einem interpersonellen Trauma als auch auf eine allgemeine Vulnerabilität für Substanzmissbrauch aus [141]. **Genom-weite Assoziationsstudien** deckten **signifikante Loci** für einen klinisch breit definierten Angst-Phänotypus und eine allgemeine Vulnerabilität für missbräuchlichen Substanzkonsum auf, die auch für einzelne Untertypen (**Alkoholabhängigkeit-Angst**: 9q33.1–q33.2, LOD = 3,054; **Drogenabhängigkeit-Angst**: 18p11.23–p11.22, LOD = 3,425) bestätigt werden konnten [79]. GWAS-Analysen im Rahmen des Psychiatric Genomics Consortium ermittelten mit der Methode des **Kopplungsungleichgewichts** (**cross-trait linkage disequilibrium (LD) score regression**) über je große Samples von PTBS (*N* = 174.659) und Alkoholabhängigkeit (*N* = 38.686) signifikante Korrelationen von weiteren gemeinsam geteilten Genorten [171].

**Zusammenfassend** ist festzuhalten, dass die Erforschung von gemeinsam geteilten epigenetischen Einflüssen auf die **Komorbidität von PTBS und Substanzkonsumstörungen** derzeit in ersten Anfängen steht [20].

## Persönlichkeitsdimensionen als gemeinsame Basis von PTBS und Substanzkonsumstörungen

Die empirische Forschung zeigt, dass die Persönlichkeitscharakteristika von Individuen auch bedeutsam genetisch mitdeterminiert sind. Genetische Einflüsse prägen in Interaktionen mit einschneidenden psychosozialen Lernerfahrungen insbesondere während der frühen Entwicklungsjahre grundlegende Dimensionen der Persönlichkeit [129]. Bestimmte **Persönlichkeitsmerkmale** und **Verhaltenstendenzen** sind als **behaviorale Endophänotypen** geeigneter, die komplexen, vielfach vermittelten Zusammenhänge von Genetik/Epigenetik und definierten psychischen Störungen für eine klinische Perspektive zu veranschaulichen [66]. Dies trifft auch auf die Komorbidität von PTBS und Substanzkonsumstörungen zu. Mehrere Persönlichkeitsdimensionen werden als Mediatorvariablen für diesen Zusammenhang diskutiert [121]:

- **Impulsivität** beschreibt eine Verhaltenstendenz, auf einen emotionalen oder motivationalen Reiz hin ohne sorgfältige Evaluation und Planung riskant, vorschnell und situationsinadäquat zu handeln. Impulsivität ist ein stark genetisch determiniertes Merkmal und ist sowohl bei Patienten mit PTBS als auch mit Substanzkonsumstörungen in besonders belasteten Subgruppen nachweisbar. Impulsivität ist mit einer niedrigen Dopamin-Baselineaktivität im Nucleus accumbens auf neutrale Reize, aber mit einer überschießenden striatalen dopaminergen Antwort auf prominente emotionale Reize und einer reduzierten präfrontalen Aktivierung assoziiert [41, 107]. Mit Impulsivität eng verwandte psychologische Konstrukte sind **Reagibilität auf Hinweisreize** („cue reactivity“) oder **emotionale Dringlichkeit** („emotional urgency“). Patient*innen mit der Dualdiagnose von PTBS und Drogenkonsumstörungen (v. a. Kokain) zeichnen sich in diesen Konstrukten durch hohe Werte aus [157, 175].
- **Angstsensitivität **ist eng mit einer subjektiven Bedrohungswahrnehmung zu verknüpfen. Sie verweist auf eine genetisch fundierte besondere Furcht vor Symptomen einer allgemeinen physiologischen Erregung [166]. Sie geht mit kognitiven Erwartungstendenzen einher, die Unheil und Bedrohung ankündigen. Sie führt zu einer Katastrophisierungshaltung und bedingt ein ausgeprägtes Vermeidungsverhalten [12]. Angstsensitivität ist ein kognitiv-affektives Brückenkonzept, das sowohl für Substanzkonsumstörungen als auch für PTBS von empirisch nachgewiesener Relevanz ist [196].

**Zusammenfassend** sind beide behaviorale Endophänotypen von Impulsivität, Reagibilität auf Hinweisreize, emotionale Dringlichkeit einerseits und Angstsensitivität andererseits als Brückenkonzepte zu verstehen. Sie liegen sowohl **externalisierenden** als auch **internalisierenden** Störungen zugrunde, die in einer entwicklungspsychopathologischen Perspektive zwei polare Vorläufersyndrome von PTBS und Substanzkonsumstörung gleichermaßen bilden [209].

## Stressoren/Traumata in der frühen Entwicklung als gemeinsame Basis von PTBS und Substanzkonsumstörungen

Multiple Stressoren und Traumata während der ersten Entwicklungsjahre haben sich epidemiologisch als wichtige Prädiktoren für den seelischen und körperlichen Gesundheitsstatus im Erwachsenenalter erwiesen. Hierbei begründen zahlreiche Gen-Umwelt-Interaktionen grundlegende Risikofaktoren (im Zusammenspiel mit protektiven Faktoren), die sowohl über allostatische Anpassungen der psychobiologischen Reaktionssysteme des Organismus als auch über gebahnte Verhaltensstile (Essen, Bewegung, Lifestyle, Alkohol, Drogen) zu intermediären Risikozuständen führen und schließlich in definierte psychische Störungen und somatische Krankheiten einmünden können [93]. Diese komplexen Entwicklungszusammenhänge in der Perspektive der Lebenszeitspanne sind sowohl für die PTBS als auch für Substanzkonsumstörungen empirisch gesichert nachgewiesen [115, 185, 200]. **Allgemein** ist für eine **Koexistenz/Komorbidität** von **PTBS und Substanzkonsumstörung** hervorzuheben:

- Effekte früher chronischer Stressoren und Traumatisierungen betreffen grundlegende Veränderungen auf allen Analyseebenen, die von der zellulären Signalgebung bis zur Verhaltensexpression reichen. Sie schließen eine Vielzahl von **Neurotransmittersystemen**, Mechanismen der Stressvermittlung wie **HPA-Achse** (dysfunktional: „Hyperkortisolismus“, „Hypokortisolismus“), **Neuroinflammation **(chronic low inflammation), **autonomes Nervensystem** (Dysbalance zwischen dominanten sympathischen und reduzierten parasympathischen Einflüssen) und zahlreiche **neuronale Regelkreise** des Gehirns ein. Einzelne betroffene Hirnregionen weisen je eigene Reifungs- und Entwicklungspfade auf und sind wiederum für eine Unzahl von distinkten Verhaltensformen verantwortlich mit ebenfalls eigenständigen weiteren Entwicklungspfaden. Einige Gehirnareale kodieren traumatische Informationen, die erst später zu Verhaltensauffälligkeiten führen. Bedeutsam erscheint in diesem Zusammenhang, dass definierte neuronale Strukturen, die im Erwachsenenalter eine zentrale Rolle in der Vermittlung traumatischer Erfahrungen spielen, wie insbesondere Amygdala, Hippokampus, und vor allem präfrontaler Kortex in frühen Abschnitten der Entwicklung noch höchst unreif sind. Frühe Traumatisierungen können aber die weitere Entwicklung dieser Strukturen in ihrer Funktionalität signifikant bahnen. Ihre potenziell pathogenen Auswirkungen werden erst sehr viel später als **atypische Stressreaktionen** erkennbar [186, 187].
- Traumata in diesen frühen Entwicklungsabschnitten betreffen in erster Linie das **neuronale Bindungssystem**. Dieses ist evolutionär so angelegt, dass es ein Baby inhärent dazu drängt, quasi unter allen, selbst unter traumatischen Umständen einen stabilen Kontakt zu einer Bindungsfigur aus Überlebensgründen aufrecht zu erhalten. Auch höchst widrige, schmerzhafte Erfahrungen werden in diese primäre Beziehungsform mit eingebunden. Von Geburt an steht eine reichhaltige **noradrenerge Neurotransmission** (Locus coeruleus) für diese basalen Lernvorgänge zur Verfügung. Die strukturelle und funktionelle Unreife jener Gehirnstrukturen, die in späteren Entwicklungsabschnitten ein Vermeidungsverhalten organisieren, wie insbesondere die Amygdala, verhindert zu diesem frühen Zeitpunkt eine effiziente behaviorale Strategie, sich von einer traumatisierenden Bindungsfigur zurückziehen zu können. Im Gegenteil, die Beziehung zu ihr ist trotz niedriger Qualität der elterlichen Fürsorge und wiederholter traumatischer Einwirkungen sogar besonders robust. Trotzdem sind jene Systeme, die in frühen Entwicklungsabschnitten noch weitgehend unreif sind, in ihrer Vorprägung von großer Tragweite in späteren Phasen der Reifung und Entwicklung [139]. Sie werden prominent zu Zeiten demaskiert, in denen weitere Stressoren oder Traumata einwirken. Mit einem **überaktiven System der Bedrohungswahrnehmung** und -**bewertung** (kortiko-amygdalär), einem bedeutsam **reduzierten Belohnungssystem** (kortiko-basalganglionär) und einem stark **eingeschränkten höher-kortikalen Kontroll- und Exekutivsystem** (präfrontaler Kortex) werden nicht nur Vulnerabilitäten aus der frühen traumatische Entwicklungsgeschichte hinsichtlich weiterer Stressoren und Traumata in spätere Lebensabschnitte transportiert, es werden auch die Chancen zu deren erfolgreicher Verarbeitung drastisch verringert [112].
- Die Konsequenzen von **Bindungstraumata** für eine besondere **Vulnerabilität zu einem suchtfördernden Verhaltensstil** sind in rezenten Übersichten zusammengestellt worden. Sowohl auf molekularer, neuroendokriner und behavioraler Untersuchungsebene lassen sich entscheidende Veränderungen im **dopaminergen System von Belohnung und behavioraler Habitbildung**, im **Oxytocin-gesteuerten Bindungssystem** sowie im **Glukokortikoid-vermittelten Stresssystem** markieren. Diese Veränderungen können sich bei unterschiedlichen Mustern einer beeinträchtigten Bindung differentiell auf prominente neurobiologische und behaviorale Aspekte auswirken, wenngleich im klinischen Kontext große Überlappungen vorkommen können [2, 98, 182]:
  - **Frühe Vernachlässigung** geht mit einer reduzierten Expression von DA-Rezeptoren und reduzierten Dopaminproduktion einher. Hierzu ist ein stark verringertes Belohnungsempfinden im ventralen Striatum grundlegendes Korrelat. In einer Perspektive der psychopathologischen Vulnerabilität sind eine von Anhedonie geprägte Depression, ein erhöhtes „sensation seeking“ mit riskanten Verhaltensstilen sowie eine erhöhte Disposition für einen Konsum von Stimulantien (Kokain, Amphetamine, Alkohol, Nikotin) hervorzuheben.
  - **Unsichere Bindung** ist mit einer reduzierten Expression von Oxytocin-Rezeptoren und reduzierten Oxytocinproduktion assoziiert. Eine Sensibilisierung gegenüber sozialen und interpersonalen Reizen mit veränderten neuronalen Aktivitäten im präfrontalen Kortex und Hypothalamus ist kennzeichnend. Psychopathologisch sind eine verstärkte Neigung zu sozialer Isolation, ein gestörtes Bindungsverhalten, ein prominenter emotionaler Schmerz sowie eine Disposition für einen Konsum von Opiaten charakteristisch.
  - **Früher Missbrauch** ist mit einer reduzierten Expression von Glukokortikoid-Rezeptoren und erhöhten CRF-Aktivität korreliert. Veränderungen in den Stresssystemen betreffen vor allem die Aktivität der Amygdala und der HPA-Achse. Psychopathologisch imponieren verstärkte Angst- und PTBS-Symptome, Zeichen eines ungelösten Traumas (komplexe PTBS) sowie eine Disposition für einen Konsum von tranquilisierenden Substanzen (Alkohol, Cannabis, Benzodiazepine, etc.).

**Zusammenfassend** tragen all diese akzentuierten neurobiologischen und behavioralen Profile als Resultanten eines früh gestörten Bindungsverhaltens zu einem allgemein und differentiell erhöhten Suchtrisiko bei. Diese früh erworbenen neurobiologischen und behavioralen Dispositionen kommen auch während der Jugend und im Erwachsenenalter im Kontext eines unsicheren Bindungsverhaltens zum Tragen. Sie können unter ungünstigen psychosozialen Entwicklungsbedingungen oder belastenden Lebenssituationen weiter verschärft werden und so zu einem Circulus vitiosus von Trauma-Sucht-Trauma-Sucht führen.

## Schlussbemerkungen

Trauma, PTBS und Sucht definieren komplexe Herausforderungen in der Versorgung. Ihre häufige Koexistenz führt zu schwerwiegenderen akuten klinischen Symptombildern mit zahlreichen, oft notfallmäßigen Hospitalisierungen. Sie trägt zu dramatisch ungünstigeren Verläufen auf allen biopsychosozialen Ebenen bei. Ein differenziertes neurobiologisches, psychologisches und soziales Verständnis der Komorbidität von PTBS und Substanzkonsumstörungen ist entscheidende Voraussetzung für eine vorteilhaftere Behandlungspraxis. Diese ist derzeit mehrheitlich noch durch getrennte Behandlungsansätze für PTBS zum einen, für Substanzkonsumstörung zum anderen mit je niedrigen therapeutischen Ansprechraten und hohen Abbruchquoten gekennzeichnet. Eine systematische und simultane Beachtung der je besonderen Ansprüche aus beiden Störungen ist in einem spezialisierten Therapiesetting zu fordern. Ein solcher integrativer Behandlungsansatz wird sich sowohl auf empirisch erprobte Trauma-psychotherapeutische Verfahren stützen und gleichzeitig innovative pharmakologische Strategien zu Therapie, Substitution und Prophylaxe der koexistenten Sucht einsetzen [6, 8, 174, 195].
